# Breast cancer insights from Northern Israel: a comprehensive analysis of survival rates among Jewish and Arab women

**DOI:** 10.3389/fonc.2024.1337521

**Published:** 2024-04-24

**Authors:** Omar Badran, Salvatore Campisi-Pinto, Mahmoud Abu Amna, Ilit Turgeman, Samih Yosef, Gil Bar-Sela

**Affiliations:** ^1^ Department of Oncology, Emek Medical Center, Afula, Israel; ^2^ Research Authority, Emek Medical Center, Afula, Israel; ^3^ Technion Integrated Cancer Center, Faculty of Medicine, Technion, Haifa, Israel

**Keywords:** breast cancer, ethnicity, Socioeconomic status, comorbidity, age-related disparities

## Abstract

This study investigates breast cancer survival rates between 2000 and 2022 in northern Israel, focusing on ethnicity, socioeconomic status, age at diagnosis, and the Charlson Comorbidity Index. Analyzing data from Clalit Health Services, we studied 8,431 breast cancer patients (6,395 Jewish, 2,036 Arab). We compared five- and ten-year survival rates across different demographics. Ethnicity showed a minor impact on survival (OR 1.12, 95% CI: 0.93 - 1.35). Socioeconomic status had a significant effect, with a higher level of improving survival (OR 2.50, 95% CI: 2.04 – 3.08). Age was crucial; women 18-39 had better survival than 60-100, but no significant difference was found between the 18-39 and 40-59 age groups [OR (CI 0.90 – 1.53, p = 0.231)]. For the Charlson Comorbidity Index, women with scores of 3-10 showed lower survival compared to scores of 0 and 1-2. There was a notable improvement in five-year survival rates among patients aged 18-59 diagnosed from 2009-2018 (90.7%) compared to 2000-2008 (86.9%) (p = 0.0046), but not in patients aged 60-100. The study highlights that socioeconomic status, age, and comorbidity scores are significant in determining survival rates for breast cancer. The improvement in survival rates for younger patients diagnosed more recently reflects advancements in treatment and care. This research provides valuable insights into the factors affecting breast cancer survival rates, underscoring the role of socioeconomic status, age, and comorbidities while also highlighting the progress in breast cancer treatment over recent years.

## Introduction

Breast cancer is a formidable adversary in the ongoing global efforts against cancer. The most diagnosed cancer worldwide (1), it has spurred extensive research efforts and evoked profound concern and awareness among women of diverse backgrounds. Breast cancer incidence is a pressing issue in Israel, a nation characterized by its diversity and complex tapestry of ethnicities (2), According to our Ministry of Health guidelines, women aged 50-74 are recommended to undergo mammography every two years, and women aged 40 and older who are at higher risk due to a family history of breast cancer or benign breast conditions are advised to undergo yearly mammography screenings (3). In contrast, the biology of the disease and the risk factors have been extensively explored. Still, gaps remain in understanding the socio-economic (4, 5) and ethnic (6, 7) disparities associated with breast cancer survival.

The intricate interplay between socio-economic status (SES), ethnicity, and health outcomes has long been a subject of intense debate and research (8–12). Socioeconomic inequalities, often mirrored by differences in access to healthcare, lifestyle, and education, can significantly influence disease prognosis and survival rates (13–17); as we know, maintaining a healthy weight is known to be crucial for reducing the risk of breast cancer. Conversely, alcohol consumption has been linked to an increased risk, while incorporating a diet rich in fruits and vegetables may lower it (18). Adhering to a balanced and nutritious diet plays a pivotal role in influencing both the risk of breast cancer onset and the subsequent outcomes following diagnosis (19). In addition, ethnicity may introduce genetic, cultural, and behavioral variations that further accentuate these disparities (20, 21). Within Israel’s unique milieu, where Jewish and Arab populations coexist and share healthcare facilities yet maintain distinct cultural and social practices, differences in survival post-breast cancer diagnosis have not been investigated. Moreover, while age and co-morbidities are widely accepted as critical determinants of breast cancer outcome (22–24), their interactions with SES and ethnicity, especially in the context of breast cancer in Israel, demand a more in-depth exploration.

The Northern District of Israel has a population of approximately 1,571,100 residents. This region stands out for its diverse demographic composition, reflecting a microcosm of the broader Middle Eastern mosaic. Jewish residents comprise 43% of the population, totaling around 603,400 individuals. This Jewish community encompasses various ethnic backgrounds, contributing to the area’s cultural richness. Nearby, the Arab community constitutes 54% of the district’s population, accounting for 755,800 residents. The Arab population adds a distinct layer of diversity, encompassing various cultural traditions and social dynamics (25). This unique blend of ethnicities, age groups, and socioeconomic backgrounds in the Northern District forms a fascinating backdrop for exploring breast cancer survival rates. It prompted us to delve deeper into the potential influences of these demographic factors on health outcomes, including age, ethnicity, socioeconomic status, and comorbidities. Additionally, we explored the outcome disparity between 2000-2008 and 2009-2016, categorized by age groups, to provide a comprehensive understanding of Israel’s evolving breast cancer landscape.

## Methods

### Data collection and processing

Israel has four health maintenance organizations (HMOs): Clalit, Maccabi, Meuhedet, and Leumit (26). Every resident is required to be insured with one of these HMOs. Approximately 70% of the population in the Northern District is certified by Clalit Health Services (CHS), the largest healthcare provider in the country (27). We analyzed the medical records of individuals in the North District of Israel with breast cancer who were members of CHS and were diagnosed in 2000. CHS members are representative of the Israeli population and reflect all demographic, ethnic, and socioeconomic groups and levels (28). CHS records are automatically collected and updated monthly in the databases of all CHS medical facilities nationwide. The data were coded, pseudonymized, viewed, stored, and processed within the CHS research room virtual platform, which only authorized researchers can access. CHS uses the International Classification of Diseases, Ninth Revision (ICD-9), and Clinical Modification to classify and index patients’ diagnoses.

#### Ethnicity

Medical outcomes were grouped according to patients’ ethnicity, primarily Jewish or Arab.

#### Age groups

To facilitate a structured analysis, the participants were grouped by age into three primary categories: young adults (18-39 years), middle-aged adults (40-59 years), and senior citizens (60-100 years).

### Changes in breast cancer survival over time

To examine changes in breast cancer survival over time, we compared 2000-2008 and 2009-2018. The first period served as our baseline, representing early 21st-century breast cancer management, and the second represented those treated according to more contemporary developments. We analyzed diverse age groups in these periods. Statistical tools helped quantify survival disparities. This comparison provides insight into breast cancer’s evolving landscape and impact on various demographics, aiding healthcare improvements.

#### Comorbidity indexing

Our data collection prioritized the health metrics of the participants, with a particular focus on their comorbidities. We employed the Charlson Comorbidity Index (CCI) for a systematic and universally acknowledged approach. Initially introduced in 1987 by Dr. Mary Charlson and her colleagues, the CCI has since been tailored for different patient demographics and medical contexts. It serves as a pivotal tool in adjusting for comorbidities in medical research.

The primary objective of the CCI is to forecast the one-year mortality risk for patients presenting with diverse comorbid conditions. Each condition is assigned a specific score. For instance, conditions such as myocardial infarction, chronic pulmonary disease, and diabetes are allocated 1 point. Meanwhile, conditions like hemiplegia and moderate renal diseases receive 2 points. Mild or severe liver disease is scored at 3, while AIDS is assigned a score of 6.

In our dataset, the cumulative scores aid in predicting mortality risk within a year. Typically, an elevated score correlates with a heightened mortality risk. For our study, we excluded the condition ‘metastatic solid tumor,’ which otherwise carries a score of 6 in the CCI.

The participants were categorized based on their CCI scores into three distinct groups: Group 1: no comorbidities (CCI score of 0); Group 2: mild comorbidities (CCI scores of 1-2); Group 3: significant comorbid conditions (CCI scores between 3-10).

#### Socioeconomic stratification

We tapped into the robust framework established by the Central Bureau of Statistics (CBS) for Israel. CBS regularly scrutinizes the socio-economic fabric of Israel, dividing it into 201 municipalities, local councils, and fifty-four regional councils. The empirical data for these evaluations stream from multifaceted sources, with pivotal contributions from agencies like the National Insurance Institute, ministry departments, and the Population and Immigration Authority. After collating information on a range of demographic, social, and economic parameters, an incisive index is engineered to mirror the resident population’s socioeconomic gradient. Employing cluster analysis techniques, local governing bodies are grouped into three broad socio-economic categories. Influenced by the CBS index, this stratification paints an insightful picture of regional prosperity and guiding macro-level policies. Therefore, the SES was determined by demographic, social, and economic parameters and categorized as low, middle, and high.

#### Statistical methods

The strength of this study lies in its robust statistical approach, by which we meticulously dissected the data to uncover patterns and correlations that illuminate the factors influencing breast cancer survival rates. Our primary objective was to discern the impact of age, ethnicity, socioeconomic status, and comorbidities on these outcomes. To achieve this, we employed various statistical methods and tools, ensuring the rigor and validity of our findings.

### Descriptive statistics

The first stage of our analysis was to use descriptive statistics to provide an overview of the dataset. This included calculating measures such as means, medians, standard deviations, and interquartile ranges to summarize the central tendencies and dispersions within the data.

#### Inferential statistics

The crux of our investigation hinged on inferential statistics, whereby we applied a battery of tests and models to draw meaningful conclusions. Essential statistical techniques included:

- Odds Ratios (OR) estimation via logistic regression analysis: We used odds ratios to quantify the likelihood of several factors influencing breast cancer survival. This measure allowed us to compare the odds of survival between diverse groups, such as age, ethnicity, and socioeconomic strata. To assess the precision of our estimates, we set confidence intervals at a 95% level.
- Chi-Square Test: The chi-square test (at a 0.05 significance level) enabled us to evaluate the association between categorical variables, such as ethnicity and survival outcomes. We determined the significance of observed differences by calculating chi-square statistics and associated p-values.
- Standard survival analysis and Kaplan-Meier curves were implemented using R statistical software (R version 4.2.2) and RStudio Version 2023.06.2.

Five-year survival measures represent the proportion (number N and %) of people who are alive for at least five years after being diagnosed with cancer.

## Results

Age and its Profound Impact on Breast Cancer Prognosis:

Our research compared different age brackets to discern patterns in survival rates. The younger cohort, aged 18-39, known for their biological resilience, was established as our baseline, mainly due to their inherent cellular vigor often highlighted in the medical literature.

The data for the middle-aged group, falling within the 40-59 years bracket, was particularly revealing. Contrary to expectations, their OR was similar to that of the younger cohort. Specifically, their OR was within a confidence interval (CI) of 0.90 to 1.53, as detailed in **Table 1**, with a p-value of 0.231. This unexpected similarity suggests that middle-aged individuals have survival patterns similar to the younger group despite the onset of age-related physiological changes. In contrast, the elderly group, aged 60-100, had different results. As outlined in **Table 1**, their OR was 0.39, with a CI of 0.30 – 0.50. This represents a substantial 61% decrease in their five-year survival rates compared to the youngest cohort.

**Table 1:** shows the logistic regression analysis results for five-year survival.

*Predictors*	Five-year survival rates		
	*Odds Ratios*	*CI*	*p*
SES Low	*Reference*		
SES Medium	1.68	1.40 – 2.02	**<0.001**
SES High	2.50	2.04 – 3.08	**<0.001**
Age (18-39)	*Reference*		
Age (40-59)	1.18	0.90 – 1.53	0.231
Age (60-100)	0.39	0.30 – 0.50	**<0.001**
Arab	*Reference*		
Jewish	1.12	0.93 – 1.35	0.221

This estimates the associations between socioeconomic status, age, and ethnicity on five-year survival rates. The bold values typically indicate statistically significant results and a key findings within the data presented.

**Figure 1:**
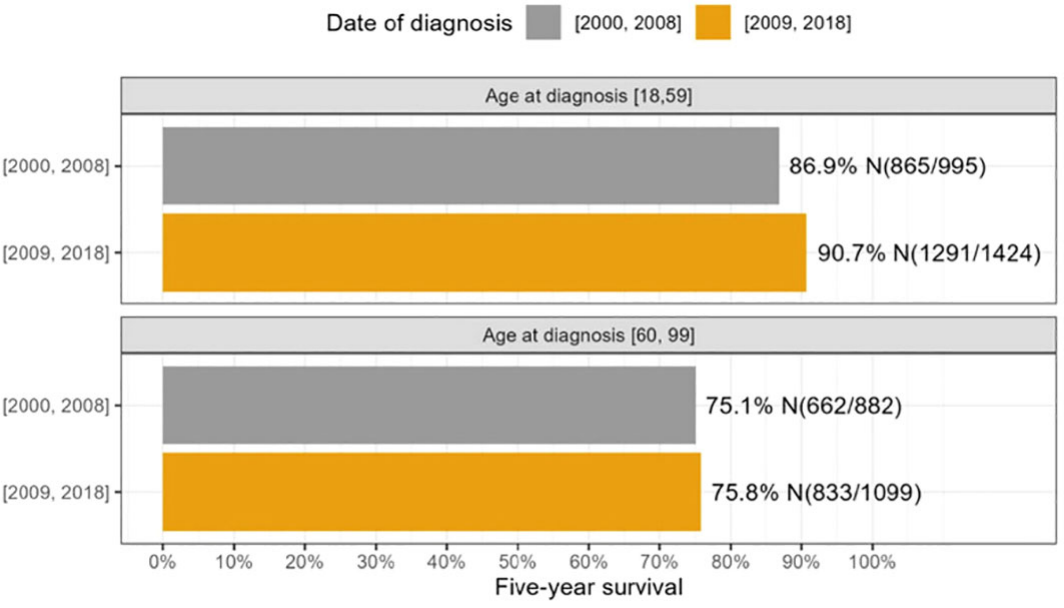
The survival differences between 2000-2008 and 2009-2018 were analyzed across age groups 18-59 and 60-100.

In addition, we investigated the 5-year survival of patients diagnosed between 2000 and 2008 compared to those diagnosed between 2009 and 2018, categorized by age groups (**Figure 1**). We found that patients in the 18-59 age group showed a statistically significant improvement in survival over this time. Specifically, those diagnosed between 2009 and 2018 had a better 5-year survival rate (90.7%) than those diagnosed between 2000 and 2008 (86.9%) (p = 0.0046). In contrast, there was no significant difference in survival between patients aged 60-100 when comparing those diagnosed between 2000-2008 and 2009-2018.

### Exploring the impact of SES on survival

Initial findings revealed a notable advantage for individuals in the middle SES category. They exhibited a 68% higher likelihood of surpassing the five-year survival milestone than their lower SES counterparts. This observation is substantiated by an OR of 1.68 [CI 1.40 – 2.02] (**Table 1**), emphasizing the potential health advantages even marginal SES improvements can confer.

Further analysis demonstrated a more pronounced advantage for those situated at the higher end of the socioeconomic spectrum. The data presented an OR of 2.50 [CI 2.04 – 3.08] (**Table 1**), signifying a significant association between elevated SES and favorable health outcomes.

**Figure 2:**
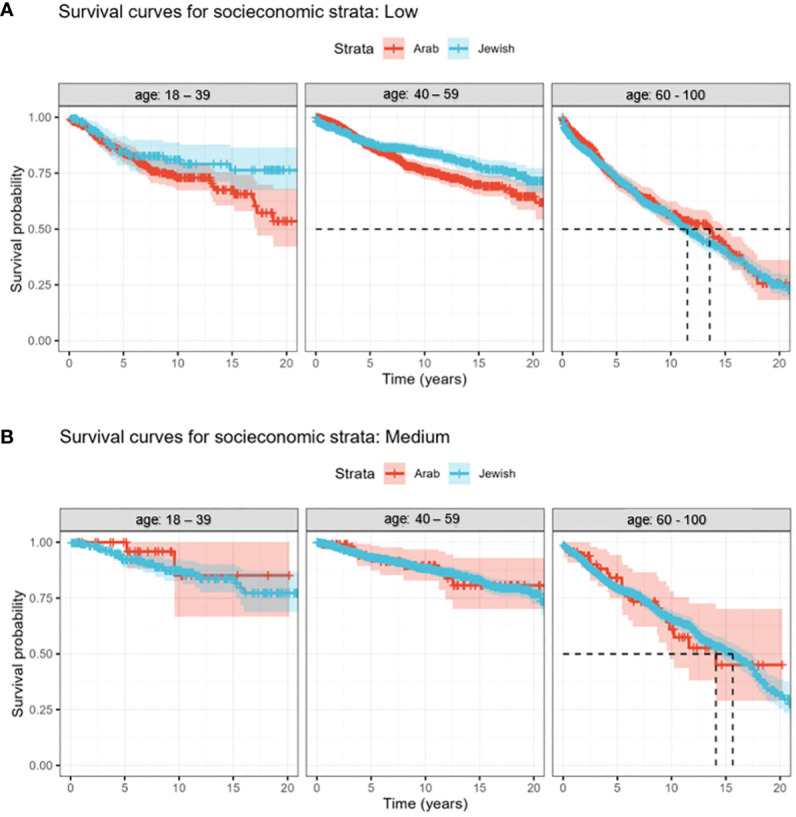
**(A, B)** Kaplan-Meier curves of the survival trends over 22 years (2000 - 2022) for Jewish and Arab populations. The study subjects are segmented into three discrete age categories. **(A)** Survival rates of breast cancer patients, segmented by age and ethnicity, within the low SES bracket. **(B)** Age-related discrepancies in survival rates among medium socioeconomic breast cancer patients.

When scrutinizing survival outcomes among women of low SES, our focus revealed negligible survival disparities between Jewish and Arab women sharing similar socioeconomic contexts. Age, however, had a marked influence on survival. A ten-year survival rate estimation unveiled a clear contrast between age groups: older women aged 60-100 exhibited a survival probability of only 50%. This stood in stark contrast to their younger counterparts, with the 18-39 and 40-59 age categories displaying an encouraging survival rate of 75% (**Figure 2A**). Consequently, ethnicity demonstrated minimal influence on survival within a low socioeconomic setting, with age prominently dictating outcomes.

Turning our attention to women in the middle SES category (**Figure 2B**), our analysis revealed consistent survival rates among Jewish and Arab populations, emphasizing the minimal role of ethnicity in these outcomes. However, age-related trends persisted, with women aged 60-100 having lower survival rates, while the 18-39 and 40-59 age groups consistently outperformed in survival metrics. Drawing parallels from the low socioeconomic bracket, this dataset reaffirmed a persistent theme: irrespective of socioeconomic standing, whether low or medium, age consistently emerged as the primary survival determinant, overshadowing ethnic distinctions.

### Ethnicity’s role in breast cancer survival outcomes

This study delved into a dataset from January 2000 to August 2017 comprising 6,187 subjects, focusing on understanding the nuances of five-year survival dynamics. The OR was 1.12 (95% CI: 0.93 - 1.35) (**Table 1**) for Jewish women’s five-year survival rate compared to Arab participants. In other words, ethnicity did not affect survival when age and socioeconomic differences were considered.

### Charlson comorbidity index: deciphering the complexity of patient health

In the study cohort, patients were systematically stratified based on their CCI scores. This stratification resulted in the delineation of three distinct groups. The first group comprised patients without reported comorbidities, as reflected by a CCI score 0. The second group included patients with mild comorbidities with a CCI score between 1 and 2. The third group encompassed patients with significant comorbid conditions with a CCI score of 3 to 10.

Our findings were multifaceted when analyzing survival rates. When juxtaposing the survival rates of the first group (CCI of 0) against the second group (CCI 1-2), we found an intriguing absence of a substantial difference in overall survival. This observation defied our initial hypothesis, which anticipated discernible variances between these cohorts.

However, the data narrative shifted profoundly upon evaluating the third group (CCI 3-10). In stark contrast to the first two groups, these patients exhibited a significant decrease in overall survival, characterized by an odds ratio of 2.4 (with a p-value of <0.001) (**Table 2**).

**Table 2:** Proportional Hazard Cox model.

Predictors	Odd Ratios	Estimate	SE	Statistic	p
Ethnicity Arab	Reference				
Ethnicity Jewish	0.924	-0.079	0.059	-1.336	0.181
Age (18- 39)	Reference				
Age (40-59)	0.845	-0.168	0.092	-1.823	0.068
Age (60-100)	2.540	0.932	0.088	10.563	>0.001
SES: Low	Reference				
SES: Medium	0.704	-0.351	0.057	-6.158	>0.001
SES: High	0.573	-0.558	0.061	-9.089	>0.001
Charlson: 0 comorbidities	Reference				
Charlson: 1-2 comorbidities	1.103	0.098	0.052	1.889	0.059
Charlson: 3-10 comorbidities	2.405	0.878	0.069	12.653	>0.001

We employed the Cox proportional hazards model to investigate age and ethnicity’s influence on survival within three distinct socioeconomic groups over 22 years (2000-2022). Standard error (SE), Socioeconomic status (SES).

### Limitations

Our study spanned two decades, from 2000 to 2022, during which diagnostic techniques, treatments, and healthcare accessibility might have evolved, all of which were not accounted for. Though broadly categorized in our research, SES has subtle nuances that could significantly influence survival rates. Cultural and behavioral differences between Jewish and Arab populations, encompassing aspects like dietary habits, lifestyle choices, or adherence to medical advice, were also not considered. These factors could have notable implications on survival outcomes. Furthermore, while the CCI is valuable, it might overlook some individual health issues or conditions pivotal to breast cancer prognosis. Lastly, the ever-present possibility remains of unmeasured confounding factors, such as genetic predispositions. Also, the breast cancer subtype cannot be extracted from the general open data repository for statistical data, as it is part of the oncology file from which data extraction is limited. This limitation underscores one of the constraints of using the database, which could further influence our reported outcomes.

## Discussion

One of the most remarkable findings from our research is the role of age as a prognostic factor in breast cancer survival rates within the Northern District of Israel. Our data intriguingly suggests that younger women have better prognoses than older women. This is interesting since the literature is divided on this issue. Some studies have found that younger women have poorer outcomes, attributing this to more aggressive tumor types and hormonal differences (29, 30). On the other hand, other research suggests that age does not significantly impact breast cancer prognosis, arguing that different age groups may have comparable outcomes (31).

It is noteworthy to emphasize that the primary cause of death in younger patients is likely to be cancer-related. This indicates that the improvements in treatment regimens and their effectiveness yield tangible results that extend the lives of these patients.

However, the situation appears somewhat different when we examine older breast cancer patients, specifically those aged 60-100. Despite the evolution of treatments and medical interventions, we did not observe a significant increase in cancer-related survival rates among this group. This discrepancy may be attributed to other factors contributing to mortality in older people, which warrant further investigation.

SES has been long recognized as a critical factor that profoundly influences health outcomes (32, 33), and our study reaffirms this connection within the specific context of breast cancer survival rates in the Northern District of Israel. However, the complex tapestry that SES requires disentangling to understand its multifaceted impact fully. Our findings point toward better outcomes for individuals from higher socioeconomic tiers, but this observation raises as many questions as it answers, necessitating further research.

Firstly, SES is more than a financial ability to afford healthcare. While economic stability undoubtedly plays a role in enabling access to quality medical care, it also often correlates with other factors, such as a better education. Higher education levels could contribute to increased health literacy, leading to earlier diagnosis and more initiative-taking healthcare behaviors, pivotal in treating diseases like breast cancer that benefit significantly from early intervention. Beyond individual or family economics, community-level socioeconomic factors may also play a role. Living in a higher-income neighborhood may mean closer proximity to well-equipped healthcare facilities and specialists, reducing the logistical burden of treatment.

Furthermore, higher SES often correlates with better social support networks. Social support’s emotional and psychological benefits have positively influenced treatment outcomes, including treatment adherence and engagement with healthcare providers (34–36), which can affect survival rates. SES also intersects with other variables explored in our study, such as age and ethnicity. For instance, the higher survival rates in younger women and the absence of ethnic disparities in survival outcomes could be attributed to underlying socioeconomic factors. Therefore, a nuanced understanding of how socioeconomic status interacts with these other variables could provide valuable insights into tailoring more effective healthcare interventions.

Our study introduces a paradigm shift in understanding the role of ethnicity in breast cancer survival rates. Contrary to prevailing assumptions and significant bodies of research that suggest race as a standalone prognostic factor (37, 38), our findings indicate that once adjusted for age and socioeconomic background, Jewish and Arab women in the Northern District of Israel had similar survival rates. This result raises several critical questions that prompt us to re-examine the conventional narrative surrounding ethnicity and health outcomes. Firstly, the absence of ethnic disparities in the study prompts questions about the role of socioeconomic and environmental factors. Does the healthcare system in Northern Israel mitigate ethnic differences observed elsewhere? It’s worth noting that There is no national standard defining the time from breast cancer diagnosis to the initiation of treatment, leaving room to establish such a measure. When the system operates smoothly, it is common for a few weeks to elapse for biopsy performance. After receiving pathology results, up to a month is needed to complete imaging, and professional discussions follow. Once a treatment plan is established, surgery or the onset of oncologic treatment typically begins within three 3-week; the health basket in Israel leads in incorporating medications for the entire population and is updated annually; for example, in the latest update, every patient who meets the criteria for the monarch E study can receive Abemaciclib therapy without additional payment from the patient (39) also there is an enhancement in the provision of diagnostic services in Israel - ranging from mobile mammography in underserved populations in the south to the deployment of equipment in large Arab villages in the north (40). Secondary ethnicity is a complex concept beyond genetics, including cultural and sociopolitical factors. These elements could influence healthcare practices and beliefs and may explain the similar survival rates observed. Thirdly, it is vital to consider the intersectionality between ethnicity and other variables, such as socioeconomic status and age. Could the absence of ethnic disparities in our study reflect more equitable social structures in the Northern District of Israel? Or is it an indicator that broader social policies aimed at reducing inequality have been successful to some extent in leveling the healthcare playing field? Additionally, this finding highlights the need for future research that dives deeper into the mechanisms through which ethnicity might impact healthcare outcomes. These could range from investigating genetic markers that may influence breast cancer survival to more qualitative research that seeks to understand how cultural practices, community beliefs, and systemic inequalities may influence health outcomes within these ethnic groups.

Our study employed the CCI as a mechanism for quantifying the burden of comorbid conditions on patients, and our findings revealed a clear, negative correlation between higher CCI scores and breast cancer survival outcomes. This echoes global research (41, 42), emphasizing the critical role of comorbidities in shaping breast cancer prognoses. However, the influence of comorbid conditions on breast cancer survival is a complex topic that merits in-depth exploration, both for its theoretical contributions to medical understanding and its practical implications for healthcare delivery. Firstly, comorbidities often impact treatment choices and outcomes (43–45). Conditions like diabetes (46), hypertension (47), and heart disease (48) can significantly affect a patient’s ability to tolerate specific treatments, such as chemotherapy or surgery. Medication interactions between medicines for comorbid conditions and breast cancer can complicate clinical care (49). This is not simply an additive effect - comorbidities often introduce a multiplicative layer of complexity to treatment protocols. For instance, diabetes complicates surgical recovery (50, 51) and can impact how well a patient tolerates chemotherapy (52, 53) and potentially influence the treatment decision of specific targeted therapies (54). Secondly, comorbidities often serve as indicators for general health and wellness, which can, in turn, affect cancer outcomes. Patients with multiple comorbid conditions may have less physiological reserve (55), poorer nutritional status (56), or more significant systemic inflammation (57, 58), impacting how effectively their bodies fight cancer and respond to treatment. Thirdly, comorbid conditions can significantly affect patients’ adherence to treatment plans (59). The more complex a patient’s medical needs, the more difficult it can be for them to manage their health effectively. This can include logistical issues, such as managing multiple medical appointments and medications, but can also involve cognitive load and psychological stress associated with managing a complex and chronic health condition alongside a cancer diagnosis.

## Conclusions

This study reveals a complex interplay of factors influencing breast cancer survival rates, in which ethnicity plays a minor role compared to SES, age at diagnosis, and comorbidities. Notably, significant improvements in survival rates emerged from 2000 to 2016, particularly among patients aged 40 to 59 years. This dynamic shift underscores the evolving landscape of breast cancer management, emphasizing the need for tailored healthcare strategies that address the multifaceted determinants of patient outcomes. These findings call for further research and intervention efforts to reduce disparities and enhance the overall prognosis of breast cancer patients.

## Data availability statement

The original contributions presented in the study are included in the article/supplementary material, further inquiries can be directed to the corresponding author/s.

## Ethics statement

The studies involving humans were approved by Omar Badran, the institutional Helsinki Review Board and data utilization committee at Haemek Medical Center, approved the use of the retrospective cohort data without requiring specific consent from the members of CHS (protocol number 003322). The studies were conducted in accordance with the local legislation and institutional requirements. Written informed consent for participation was not required from the participants or the participants’ legal guardians/next of kin in accordance with the national legislation and institutional requirements.

## Author contributions

OB: Conceptualization, Data curation, Writing – original draft, Writing – review & editing. SC-P: Data curation, Formal analysis, Validation, Writing – review & editing. MM: Validation, Visualization, Writing – review & editing. IT: Validation, Visualization, Writing – review & editing. SY: Validation, Visualization, Writing – review & editing. GB-S: Conceptualization, Supervision, Visualization, Writing – review & editing.
